# SSTR2 in Nasopharyngeal Carcinoma: Relationship with Latent EBV Infection and Potential as a Therapeutic Target

**DOI:** 10.3390/cancers13194944

**Published:** 2021-09-30

**Authors:** Oscar Emanuel, Jacklyn Liu, Volker H. Schartinger, Wen Long Nei, Yuk Yu Chan, Chi Man Tsang, Herbert Riechelmann, Liam Masterson, Johannes Haybaeck, Udo Oppermann, Stefan M. Willems, Marc L. Ooft, Guido Wollmann, David Howard, Bart Vanhaesebroeck, Valerie J. Lund, Gary Royle, Melvin L. K. Chua, Kwok Wai Lo, Pierre Busson, Matt Lechner

**Affiliations:** 1 UCL Cancer Institute, University College London, London WC1E 6BT, UK; Oscar.emanuel@nhs.net (O.E.); regmjwj@ucl.ac.uk (J.L.); bart.vanh@ucl.ac.uk (B.V.); v.lund@ucl.ac.uk (V.J.L.); g.royle@ucl.ac.uk (G.R.); 2 Department of Otorhinolaryngology, Medical University of Innsbruck, 6020 Innsbruck, Austria; volker.schartinger@i-med.ac.at (V.H.S.); herbert.riechelmann@i-med.ac.at (H.R.); 3 National Cancer Centre, Divisions of Radiation Oncology and Medical Sciences, Singapore 169610, Singapore; nei.wen.long@singhealth.com.sg (W.L.N.); melvin.chua.l.k@singhealth.com.sg (M.L.K.C.); 4 Oncology Academic Programme, Duke-NUS Medical School, Singapore 169857, Singapore; 5 Department of Anatomical and Cellular Pathology, The Chinese University of Hong Kong, Hong Kong 999077, China; ellischan@link.cuhk.edu.hk (Y.Y.C.); annatsang@cuhk.edu.hk (C.M.T.); kwlo@cuhk.edu.hk (K.W.L.); 6 State Key Laboratory of Translational Oncology, The Chinese University of Hong Kong, Hong Kong 999077, China; 7 Department of Otolaryngology, Addenbrooke’s Hospital, Cambridge CB2 0QQ, UK; liam.masterson@ad-denbrookes.nhs.uk; 8 Institute of Pathology, Neuropathology and Molecular Pathology, Medical University of Innsbruck, 6020 Innsbruck, Austria; johannes.haybaeck@i-med.ac.at; 9 Botnar Research Centre, University of Oxford, Oxford OX1 2JD, UK; udo.oppermann@ndorms.ox.ac.uk; 10 Freiburg Institute for Advanced Studies (FRIAS), University of Freiburg, 79085 Freiburg, Germany; 11 Department of Pathology, University Medical Center Utrecht, 3584 CX Utrecht, The Netherlands; s.m.willems@umcg.nl (S.M.W.); m.ooft@nhs.net (M.L.O.); 12 Department of Pathology, University Medical Center Groningen, 9713 GZ Groningen, The Netherlands; 13 King’s College Hospitals, NHS Foundation Trust, London SE5 9RS, UK; 14 Institute of Virology and Christian Doppler Laboratory for Viral Immunotherapy of Cancer, Medical University of Innsbruck, 6020 Innsbruck, Austria; guido.wollmann@i-med.ac.at; 15 ENT Department, Charing Cross Hospital, Imperial College Healthcare NHS Trust, London W6 9EP, UK; davidjhoward10@gmail.com; 16 Royal National Throat, Nose and Ear Hospital, University College London Hospitals NHS Trust, London WC1E 6DG, UK; 17 CNRS-UMR 9018-Metsy, Gustave Roussy and Université Paris-Saclay, 94805 Villejuif, France; 18 Rhinology & Endoscopic Skull Base Surgery, Department of Otolaryngology-H&N Surgery, Stanford University School of Medicine, Palo Alto, CA 94305, USA

**Keywords:** nasopharyngeal carcinoma (NPC), somatostatin receptor 2 (SSTR), EBV, epidemiology, global health, biomarkers, carcinogenesis, imaging, therapeutics

## Abstract

**Simple Summary:**

Nasopharyngeal cancer (NPC) is a malignant epithelial tumor endemic to parts of Asia and associated with infection by the Epstein–Barr virus (EBV) in these regions. The cancer is often detected at a late stage which is associated with poor outcomes (63% 5-year survival). Advances for the management of this disease have remained largely stagnant and treatment relies primarily on radiotherapy and chemotherapy, as well as surgery when indicated. Nevertheless, our understanding of its underlying biology has grown rapidly in the past two decades, laying the foundation for the development of improved therapeutics which have the potential to improve outcomes. This review offers a comprehensive, up-to-date summary of this disease, with a focus on the role of somatostatin receptor 2 (SSTR2) in NPC and how this increased knowledge may lead to improved diagnosis and management of this disease.

**Abstract:**

Nasopharyngeal carcinoma (NPC) is a malignant epithelial tumor, most commonly located in the pharyngeal recess and endemic to parts of Asia. It is often detected at a late stage which is associated with poor prognosis (5-year survival rate of 63%). Treatment for this malignancy relies predominantly on radiotherapy and/or systemic chemotherapy, which can be associated with significant morbidity and impaired quality of life. In endemic regions NPC is associated with infection by Epstein–Barr virus (EBV) which was shown to upregulate the somatostatin receptor 2 (SSTR2) cell surface receptor. With recent advances in molecular techniques allowing for an improved understanding of the molecular aetiology of this disease and its relation to SSTR2 expression, we provide a comprehensive and up-to-date overview of this disease and highlight the emergence of SSTR2 as a key tumor biomarker and promising target for imaging and therapy.

## 1. Introduction

**Figure 1:**
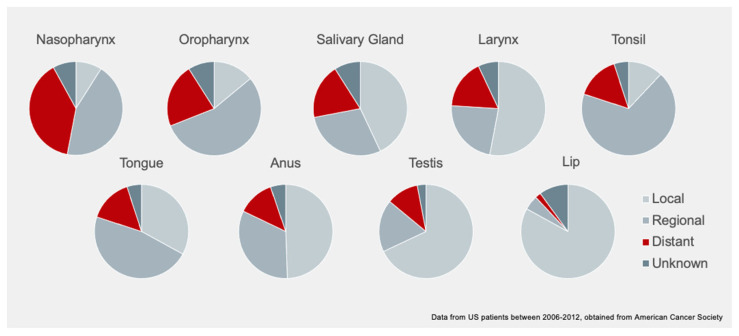
Stage distribution pattern of selected cancers at presentation, including tonsil, oropharynx, tongue and others, which have a similar geographical distribution as NPC, with higher rates where NPC is endemic.

Nasopharyngeal cancer (NPC) is a malignant epithelial head and neck cancer that occurs most frequently in the pharyngeal recess (fossa of Rosenmuller) of the nasopharynx and is endemic in Southeast Asia where most cancers are associated with Epstein–Barr virus (EBV) infection. Patients often present at a late stage and Figure 1 illustrates data gathered from US patients from 2006–2012 by the American Cancer Society, detailing stage distribution of adult head and neck cancers at presentation [1]. This is in line with the poor prognosis, and frequent recurrences and metastases in NPC. The median overall survival falls between 11 and 28% in recurrent and metastatic cases, with a median survival range of 12 to 24 months [2,3]. The 5-year overall survival for stages I, II, III, IVA and IVB NPC was 93%, 86%, 65% and 63%, respectively, in a meta-analysis of 3328 Hong Kong patients over 10 years [4].

It was shown that EBV infection upregulates the expression of the somatostatin receptor 2 (SSTR2) [5]. SSTR2 is a G protein-coupled receptor (GPCR), encoded by a gene located on the long arm of chromosome 17. SSTR2 is most highly expressed in the alpha and beta cells of the pancreas, where it has a specific inhibitory effect on pancreatic enzyme secretion when bound by somatostatin, and is also found in the cerebrum, kidney, jejunum, colon and liver [6,7].

SSTR2 is a well-established clinical biomarker of neuroendocrine tumors (NETs) of the gut, including pancreatic NETs, where its consistent overexpression was exploited for the management of these malignancies through targeted imaging and therapeutics. In two recent works, SSTR2 overexpression was demonstrated in NPC and, strikingly, shown to be significantly enriched in EBV-associated disease [5,8]. In order to understand the relevance of SSTR2 to EBV-related NPC, it is important to have an understanding of the relationship between EBV and NPC, including potential signaling pathways such as the NF-κB pathway that drives SSTR2 expression within NPC cells. Here, we present a comprehensive and up-to-date review of EBV-associated NPC with a discussion on pathways of oncogenesis, including contributions by viral and cellular factors in order to appreciate the effects of EBV infection on both. We also focus on the role of SSTR2 in NPC and explore exciting new diagnostic capabilities, imaging modalities and treatment options. We conclude the review with a proposed treatment protocol which we hope might stimulate interest in and debate about future clinical decision-making in EBV-associated NPC.

## 2. Non-Viral Risk Factors

### 2.1. Environmental Risk Factors

The association of EBV with NPC is well-established [9]. Documented risk factors for NPC include the consumption of preserved foods, especially when given to young children when weaning, which may, among other factors, account for the endemic geographical patterns observed. One classical historical example is salted fish consumed by the Hakka people, who are an ethnic group residing in the estuaries of Southern China [10]. Diets with poor fruit and vegetable intake are also risk factors. The temporal trend of the decreasing incidence in NPC observed in the last 20 years in large cities with prevalent Chinese populations, such as Hong Kong and Singapore, may well be linked to reduced consumption of preserved food, especially in young children. These environmental or dietary factors correlate with the finding that second-generation Chinese born in the West have a lower risk of developing NPC compared to first-generation Chinese immigrants in the same countries, although increasing genetic diversity may also play a role [11,12]. It is important to note, however, that the incidence of NPC in China is highly heterogenous with record incidence rates in the south of the country and some of the lowest rates in the world in the northern part. For this reason, China comes 14th worldwide, with an incidence of 3.0 per 100,000, while Brunei has the highest incidence, 9.9. Figure S1 at the end of this article was created using data from the World Cancer Research Fund to compare the incidence rates per 100,000 population against the incidence rates not adjusted for population in the countries with the highest incidence of NPC.

An estimated 23% of NPC cases are attributable to smoking tobacco while alcohol consumption was also shown to contribute to carcinogenesis [13]. Cannabis was also reported to represent a risk factor [14]. Interestingly, the relative risk is increased in EBV-positive NPC patients with occupational exposure to wood dust and formaldehyde, which themselves were reported to represent risk factors for developing NPC [13]. A similar association was seen in NPC patients who smoke tobacco. No such correlation was found between regular alcohol intake and cancer predisposition in EBV-positive NPC [15]. In summary, such as in other cancers, NPC carcinogenesis is multifactorial with a combination of environmental risk factors potentiating carcinogenesis.

### 2.2. Host Genetic Susceptibility

Host genetic susceptibility plays an important role in the development of NPC, at least in a subset of cases. Patients are at a much higher risk of EBV-associated NPC if there is already one case in their ascendants or siblings. With its viral aetiology, it is unsurprising that early linkage studies found significant associations between various HLA alleles and increased risk of NPC [16,17,18]. Over the past decade or so, a handful of genome-wide association studies (GWAS) were conducted, which allow for the identification of susceptibility loci across the genome. GWAS conducted by Tse et al., Bei et al. and Chin et al. reiterated the importance of polymorphisms in the MHC region with regards to NPC risk [19,20,21]. In their GWAS of 277 Taiwanese NPC patients, Tse et al. identified 12 statistically significant SNPs, all of which mapped to 6p21.3, two of which were located within the *HLA-A* gene. The association of *HLA-A* to NPC was further confirmed by Chin et al. in their cohort of 184 Malaysian Chinese NPC patients, with the strongest association found with rs3869062. Lastly, Bei et al. demonstrated multiple associations within the MHC region, including rs2860580, rs2894207 and rs28421666 in their assessment of 1583 Chinese NPC patients. However, in contrast to Chin et al., an association within the *ITGA9* gene was not observed. Furthermore, associations within the previously identified susceptibility loci 3p21, 4p15.1-q12 and 5p13 were not detected. Genetic heterogeneity as well as differences in sample size between study groups likely account for this discrepancy. Lastly, a recent large-scale whole-exome sequencing (WES) study of 5689 Hong Kong Chinese identified eight independent signals with six located at *HLA-A/B* and *HLAα chain 1* alongside validation of previously reported findings by GWAS. This fine-mapping analysis of a large cohort, which overcomes shortcomings encountered in previous studies, provides further support for the importance of HLA in the aetiology of NPC [22].

There are also genetic associations outside the MHC region. In two case-controlled studies, increased NPC risk was observed in those patients with a polymorphism in *CCND1*, which encodes the cell-cycle regulator cyclin D1, as well as *TLR3*, which encodes a toll-like receptor belonging to the family of pattern recognition receptors involved in innate immunity [23,24]. Additionally, across the above GWAS, as well as a fourth conducted by Ng et al., associations were found within the *ITGA9*, *GABBR1*, *TNFRSF19* and *MDS1-EV11* genes. *ITGA9* encodes an integrin, which plays an important role in cell adhesion as well as cell proliferation, differentiation and malignant transformation. *GABBR1* encodes a GABA receptor and, in the corresponding study, the authors detected GABA receptor protein in their NPC biopsies and in vitro, which indicates a potential role for GABA signaling in carcinogenesis, however, this has not been further explored [19]. *TNFRSF19* and *MDS1-EV11* encode proteins involved in JNK signaling, caspase-independent cell death as well as cell proliferation.

A meta-analysis of the aforementioned four GWAS suggests a strong association with NPC of polymorphisms in rs31489 and other single-nucleotide polymorphisms located within the *CLPTM1L/TERT* region on chromosome 5p15.33 [25]. The membrane protein encoded by the *CLPTM1L* gene causes apoptosis in cisplatin-sensitive cells when overexpressed and is also associated with lung, pancreatic and breast cancers. The *TERT* gene plays an important role in telomere maintenance and enhancing telomerase activity. It has been shown to be activated by the latent membrane protein 1 (LMP1) of EBV [26]. Interestingly, a recent WES study of multiplex families identified variants in 12 genes likely involved in cancer pathogenesis, including *CLPTM1L*, as well as *NOTCH1*, *DLL3*, amongst others [27].

It is important to note that in the context of NPC, widespread susceptibility alleles, such as those linked to the HLA locus and the *TERT* gene, show a weak penetrance. There are genes with high penetrance, but these are found in small family clusters. One such gene, identified through WES, is the macrophage-stimulating 1 receptor (*MST1R*), which appears to be linked to NPC at the early age of onset. In a WES study of 161 NPC cases of Southern Chinese descent in 2016, 13 independent cases carrying the *MSTR1* pathogenic heterozygous germ-line variants were identified. Of these 13, a total of 7 (53.8%) were diagnosed with NPC at the age of 20 or younger. A subsequent validation study of 2160 cases and 2433 controls demonstrated a strong association of *MST1R* variant c.G917A:p.R306H with NPC, with an odds ratio of 9.0 [28]. Altogether, these studies affirm the multifaceted development of NPC with, in addition to viral infection, both environmental and genetic factors playing important roles in risk, potentially serving as useful targets for the earlier detection of disease. However, whether or to what extent the genes discussed above contribute to oncogenesis remains unclear.

## 3. Molecular Mechanisms of Oncogenesis

### 3.1. Virally-Mediated Oncogenesis

**Figure 2:**
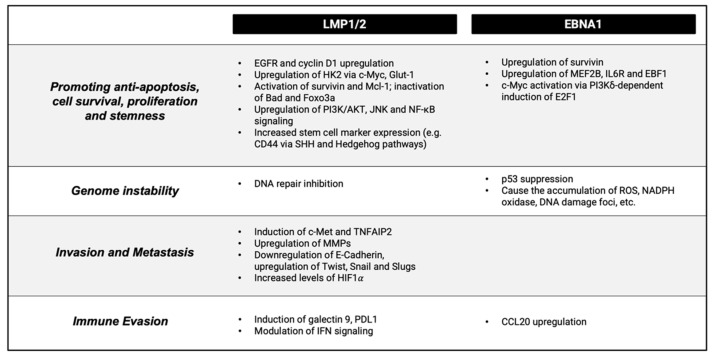
A schematic of EBV-induced NPC pathogenesis, highlighting the action of the key viral oncoproteins LMP1 and 2 and EBNA1 and their contribution to various hallmarks of cancer.

EBV infection is a key contributor to NPC development through the actions of its viral products including the LMP1 and EBNA1 proteins, the BART microRNAs and the EBER RNAs. Importantly, latent infection is largely understood to precede clonal expansion of tumor cells and plays a crucial role in the initiation of NPC carcinogenesis. The oncogenesis process and the establishment of latent infection in EBV-positive NPC differs from that of other EBV-related cancers. The virus, though able to transform and immortalise primary B cells into proliferative clones, is unable to do so for primary nasopharyngeal epithelial cells [29]. Memory B cells are transformed into proliferative lymphoblastoid cell lines (LCLs) upon EBV infection. This differs from EBV infection of normal pharyngeal epithelial cells, in which NPC pathogenesis depends on the establishment of persistent latent EBV infection, during which EBV expresses three latent membrane proteins—LMP1, LMP2A and LMP2B. LMP1 activates the NF-κB signaling pathway, which will be discussed later in this review. EBNA1, an EBV protein needed for the stable persistence of EBV episomes, is also expressed during latent EBV infection in NPC. The five other EBV-encoded nuclear antigen proteins (EBNA2, EBNA-3A, EBNA-3B, EBNA-3C and EBNA-LP) are not expressed in the context of NPC [30,31]. In NPC, persistent EBV infection can be established in a genetically aberrant epithelial cell (which will be described more in Section 3.2). EBV infection and progressive genomic changes are postulated to result in tumorigenic transformation (Figure 2) [32].

Crucially, pre-malignant changes are necessary to accommodate latent infection. This occurs through an autoregulatory loop whereby EBNA1 protein binds near its RNA initiation site to limit transcription. Latency is further driven by the *Bam*HI-Q promoter (Qp) which itself is regulated by viral and cellular factors, including NF-κB signaling [33]. Indeed, the NF-κB signaling protein, p65, can activate EBV Qp, while p65 activation is inhibited by EBNA1. However, it is uncertain whether other NF-kB signals, such as p50/BCL3 or p50/RelB, play a role in Qp activation. With this, a regulatory loop consisting of EBNA1 and NF-κB signaling to support EBV latent gene expression while limiting NF-κB activity was proposed [24]. Therefore, specific inhibition of EBNA1 is a strategy being explored to combat tumor cell growth in EBV-associated cancers. Such growth inhibition may be achieved through the inhibitory ability of dominant-negative EBNA1 and downregulation of EBNA1 expression by antisense oligodeoxynucleotides [34,35,36,37,38,39]. Inhibition of NF-κB signaling by the IκB kinase inhibitor PS-1145 results in downregulation of Qp-EBNA1 expression in cells of the EBV-positive NPC-derived cell line C666-1 [33].

EBV ubiquitously expresses microRNAs (small single-stranded non-coding *RNA* molecules, containing about 22 nucleotides) during latency. A total of 22 microRNAs (miR-BARTs) are encoded in the intronic regions of BamHI fragment A rightward transcripts (BARTs) of the EBV genome. These include: miR-BART2 and two microRNA clusters, cluster 1 (miR-BART1,3,4,5,6,15,16,17) and cluster 2 (miR-BART7,8,9,10,11,12,13,14,18,19,20,22) based on their locations on the BamHI-A region [40]. EBV *miR-BART* microRNAs target and repress cellular pro-apoptotic genes, promoting host cell survival through binding and downregulating cellular mRNAs. This offers some explanation for the relative resistance of EBV-associated NPC to DNA-damaging treatments such as chemotherapy and radiotherapy [41]. PUMA (p53 up-regulated modulator of apoptosis) is repressed by the *miR-BART5* that is overexpressed in NPC and can also be found in the context of EBV-associated gastric carcinoma cells [42].

Some EBV isolates are associated with a higher risk for NPC. Through genome-wide association studies, the *BALF2* region was found to have the greatest association among the multiple association signals along the EBV genome in NPC patients from Southern China [43]. Fine-mapping analysis was applied to identify causal SNPs (single nucleotide polymorphisms. A significant association was found only in the three non-synonymous coding variants (NC_007605.1:162215C>A, 162476T>C and 163364C>T). They identified high-risk *BALF2* subtypes (C-C-T or C-C-C) which contribute to 83% of the overall risk of NPC in Southern China cumulatively [43]. *BALF2* encodes a DNA binding protein, which is critical to EBV lytic replication. The authors postulate that these genetic changes may alter the function of the protein and subsequently dysregulate the EBV lytic cycle. However, whether these polymorphisms directly play a role in the increased oncogenic capacity of the resulting variants is uncertain and further elucidation of the underlying mechanisms is required.

Lastly, *EBER* (EBV-encoded small RNA) is the most abundant EBV viral transcript in NPC. It is responsible for the activation of TLR3 (toll-like receptor 3) signaling for inducing IGF-1 (insulin-like growth factor 1) production, which was found consistently overexpressed in NPC biopsies and shown to contribute to NPC oncogenesis by acting as an autocrine growth factor [44]. The EBV-positive cell line C666-1 is dependent on IGF-1. Furthermore, *EBERs* block cellular responses to interferons, conferring resistance to IFN-α-induced apoptosis via binding to PKR (RNA-activated protein kinase) and inhibition of its phosphorylation [45].

### 3.2. Contribution of Cellular Factors to Oncogenesis

Early cellular events in carcinogenesis involve cell cycle dysregulation and inactivation of tumor suppressor genes, which help facilitate persistent latent EBV infection. Viral factors then drive the acquisition of oncogenic cellular changes, which lead to the establishment of various hallmarks of cancer including aberrant cell growth, inhibition of apoptosis, immune evasion, angiogenesis, cellular invasion, and metastasis. This was recently reviewed in depth by Tsang et al. [46]. To summarize, EBV protein products can drive aberrant cell signaling through transcriptional regulation, perturbing pathways such as NF-κB, PI3K/AKT/ERK and Hedgehog signaling. For example, LMP-1 was shown to increase cell proliferation and inhibit apoptotic activity through its action on EGFR signaling, cyclin D1 regulation of the cell cycle, the anti-apoptotic proteins survivin and MCL-1 and the pro-apoptotic proteins Bad and Foxo3a, as well as helping to facilitate growth-inducing metabolic changes. Virally mediated changes to Hedgehog signaling allow for the acquisition of stem-like properties while upregulation of DNA methyltransferase 1 (DNMT1) leads to global hypermethylation and consequent epigenetic silencing of important tumor suppressors.

In addition to changes to cell signaling, important genetic changes include loss of 3p and 9p, which consist of a tumor suppressor gene cluster and genes encoding the cell cycle regulators p16 (*CDKN2A*) and p15 (*CDKN2B*). The subsequent loss of tumor suppressor activity, including that of *CDKN2A*, *RASSF1A* and *TGFBR2*, are considered early carcinogenic events in NPC. Amplification of chromosome 12 and frequent loss of 11q, 13q, 14q and 16q were also described. As well as p16/*CDKN2A* deletion, overexpression of cyclin D1 is a driver event in NPC and is associated with positive EBER staining in the epithelium of dysplastic nasopharyngeal epithelium, supporting its link with EBV infection in the early stages of NPC. Overexpression of cyclin D1, as well as the presence of a p16-resistant form of CDK4 (CDK4R24C) and loss of p16/CDKN2A, suppress cellular differentiation, which has implications concerning its association with EBV infection in undifferentiated NPC. In vitro cell-based models have shown that EBV-infected cells exhibit cellular growth arrest and senescence immediately after infection with EBV. This suggests the nasopharyngeal mucosa may be “set” for persistent EBV infection at that stage [47].

Several recent studies have sought to elucidate the genomic landscape of NPC, all of which have identified C>T transitions at NpCpG sites as the predominant mutational signature in NPC [48,49,50,51]. Additional significant signatures include those arising from defective homologous recombination repair, DNA mismatch repair as well as the APOBEC mutation signature. While mutational rates are suspected to be low, in their study of 111 tumor specimens, Li et al. observed a mutational burden of >50 mutations/tumor [49]. Nevertheless, across studies, mutations within key oncogenic pathways, including p53 inactivation and PI3K/AKT/mTOR signaling, as well as within genes involved in chromatin regulation, are infrequent. For example, *TP53* mutations are relatively rare, seen in roughly 8–10% of NPC despite being among the frequent gene mutations identified alongside genes such as *TRAF3*, *CYLD*, *NFKBIA* [48,49,51]. Loss-of-function mutations in the latter three genes were observed across studies. Crucially, these genes encode negative regulators of the NF-κB signaling pathway. Genes involved in PI3K/MAPK signaling are also frequently mutated. Importantly, overall low mutational rates in these and other pathways may be due to a lack of selection pressure to acquire these changes due to the ability of viral proteins to alternatively modulate their activity. Indeed, mutational changes in the NF-κB pathway are mutually exclusive of LMP-1 overexpression.

Epigenetic dysregulation of various signaling pathways, including retinoid, WNT, MAPK, TGF-β and Hedgehog signaling, and cancer-associated genes, including those encoding cadherins, matrix metalloproteinase, checkpoint regulators as well as long non-coding and microRNAs, were described in NPC (reviewed by Tsang et al. [46]). Hypermethylation of *CDKN2A*, as well as RASSF1A, are regarded as important events in carcinogenesis [52]. Recently, the role of histone modifications in the activation of NF-κB subunits, *IRF1/2* and *MYB* was demonstrated, in addition to the identification of super-enhancer targets, such as *ETV6*, as potential elements in NPC oncogenesis [53,54].

Of particular note, genetic and epigenetic alterations of *TGFBR2* were consistently demonstrated in NPC [51,55]. Stimulation of TGF-β leads to phosphorylation of SMAD2 and SMAD3 to form a complex with SMAD4 resulting in growth inhibition and cellular differentiation. As a driver event, *TGFBR2* inactivation can evade growth inhibition in NPC by abrogating SMAD-dependent TGF-β signaling. In TGFBR2 knockout nasopharyngeal epithelial cells, the number of cells with stable infection was significantly increased after EBV infection. The findings point to a co-operative role of impaired cell cycle and TGF-β signaling pathways in creating a susceptible epithelial lesion capable of supporting EBV latency during NPC transformation [51]. Constitutive activation of the NF-κB pathway is the most consistent feature of NPC cell phenotype and will be discussed later in the article.

## 4. SSTR2 and the Pathogenesis of Nasopharyngeal Carcinoma

### 4.1. Somatostatin Receptors in Cancer outside the Nasopharynx

SS-Rs are seven G protein-coupled transmembrane receptors, whose ligand is the neuropeptide somatostatin, and exist in five isoforms: SSTR1-5. SS-Rs are overexpressed in neuroendocrine tumors (NETs), two-thirds of which originate from the GI tract, including carcinoid tumors. Non-carcinoid gastroenteropancreatic tumors consist mainly of insulinomas, gastronomes and vasoactive intestinal peptide-secreting tumors. Outside the GI tract, SS-R overexpression was demonstrated in small cell lung cancer, phaeochromocytoma, neuroblastoma and medullary thyroid cancer [56]. SS-Rs are also expressed in solid tumors including prostate, breast, colon, lung, liver, renal, adrenal cortex, pancreatic adenocarcinoma and thyroid.

### 4.2. SSTR2 and Nasopharyngeal Cancer

The expression of SSTR2 in NPC was discovered in 2002 when Loh et al. examined SS-R expression in 12 NPC biopsy specimens and 5 non-neoplastic specimens. They noted moderate to high expression of somatostatin receptors in 9 of the 12 NPC specimens, specifically SSTR2, and no SS-R expression in the 5 granulomatous non-neoplastic tissue specimens [4]. SS-R expression in EBV-positive NPCs was further validated by Schartinger et al. in 2015, whose imaging of five histologically proven EBV-positive NPCs with ^68^Ga-DOTA-TOC PET/CT demonstrated tracer uptake similar to that in highly differentiated NETs [57]. Before this, radiological evidence was limited to single case reports, such as a 2008 report by Bennick et al. who performed an Indium-111 octreotide scan when attempting to distinguish between two diagnoses of meningioma (which expresses SS-R) and chordoma (which does not express SS-R) in a patient with headaches and diplopia. Tumor and lymph node biopsy led to a diagnosis of NPC which was accompanied to their surprise by nodal and primary skull base lesion octreotide uptake [58]. It is an intriguing thought that the strong uptake of 68Ga-labelled [DOTA0, Tyr3]-octreotide (68Ga-DOTA-TOC PET/CT) may be an indicator for octreotide-based radionuclide treatment. Octreotide is a synthetic somatostatin analogue used in the treatment of NETs [59]. We will look at treatment options in further detail later in this review including potential novel treatment options which may be more promising than the currently used octreotide and lanreotide. Recently, our consortium led by Lechner et al. performed immunohistochemistry on the largest published series of primary, recurrent, and metastatic NPCs to reveal that SSTR2 expression is found in 81% of primary tumor samples [5]. There was no significant difference in expression levels between cases and, interestingly, expression was maintained in locally recurrent and metastatic disease, indicating the potential broad clinical utility of SSTR2 in this setting. In this population study, patients who were negative for both EBV and SSTR2 were found to have the poorest prognosis. Exploration of the mechanism of SSTR2 expression in the context of NPC revealed that SSTR2 expression is induced by EBV via the NF-κB pathway. Notably, the EBV status alone was shown to have a less significant prognostic value when considered independent of SSTR2 status [4]. Therefore, SSTR2 could be a useful future prognostic biomarker of NPC.

## 5. The NF-κB Pathway

We have already touched on the NF-κB pathway and NF-κB signaling in NPC cells earlier when discussing both the viral and cellular factors driving NPC oncogenesis. In this section, we will provide an overview of the NF-κB pathway and its relationship to SSTR2 expression. The transcriptional factors p50, p52, RelA/p65, RelB, c-Rel and BCL3 comprise the NF-κB (nuclear factor kappa-light-chain-enhancer of activated B cells) family [60,61]. Activation of the NF-κB protein complex mediates cell proliferation, apoptosis, cell transformation and immunosuppression. Its activation has already been established outside the nasopharynx, with oncogenic effects in both solid and haematological malignancies related to its aberrant regulation [62].

**Figure 3:**
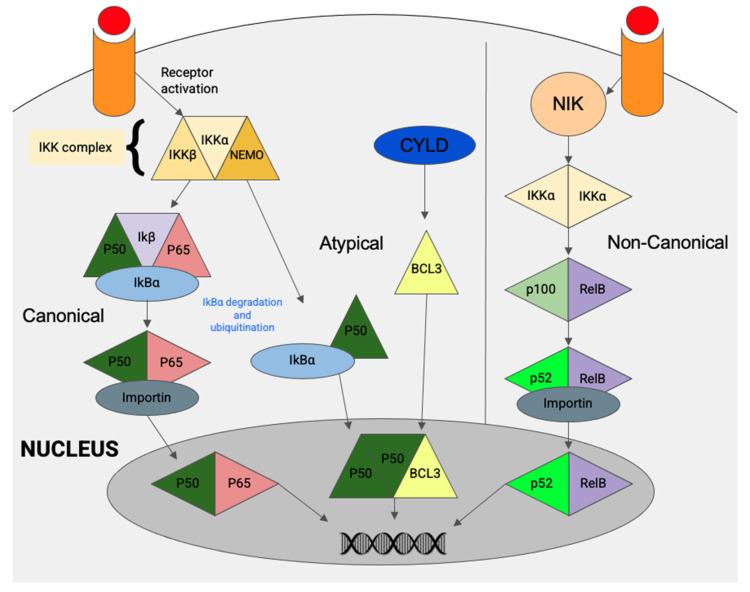
Simplified schematic of NF-κB signaling, comprised of the canonical, non-canonical and atypical pathways.

Constitutive activation of the NF-κB pathway is a consistent feature of NPC and detection of p50/p50/Bcl3 or p50/RelB (p65) was demonstrated in a cohort of NPC specimens [60]. In contrast to other cancers, NF-κB activation in NPC is mainly not of the canonical type but predominantly occurs through the atypical pathway, with detection of p50/p50/Bcl3 in up to 95% of cases (Figure 3) [46]. In NPC, persistent NF-kB pathway activation can be contributed by overexpression of LMP1 or through genetic alterations of NF-κB negative regulators (*TRAF3*, *CYLD*, *NFKBI*) and receptors (*LTBR*). It has long been known that LMP1 mediates NF-κB activation via two effector regions in its carboxy-terminal cytoplasmic domain, as demonstrated by Heun et al. in 1995 [63]. In NPC, LMP1-modulated NK-κB activation was shown to induce SSTR2 expression [5]. Furthermore, NF-κB signaling may also be activated by EBV-miR-BART13, an EBV micro-RNA which was shown to promote NPC cell growth and metastasis in both in vitro and in vivo studies [64]. Expression of the NF-κB inhibitor interacting Res-Like 2 (NKIRAS2) protein is inversely correlated with NF-κB expression due to its role as a negative regulator of NF-κB. EBV infection in the context of NPC can be seen to alter this regulation as NKIRAS2 is directly targeted by EBV-miR-BART13 in NPC cells [64].

**Figure 4:**
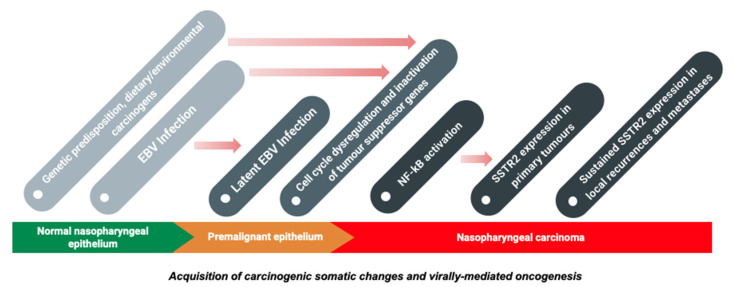
An overview of the pathogenesis of NPC and potential pathway leading to overexpression of SSTR2.

As mentioned above, the genomic aberrations of negative regulators on the NF-κB pathway were investigated in 2017 by Li et al. who performed exome and genome sequencing on EBV-positive NPCs. This identified *CYLD*, *TRAF3*, *NFKBIA* and *NLRC5* as possible responsible negative regulators in 41% of the 111 cases [49]. The study has helped to build the genomic landscape of EBV-positive NPC. Among the specimens micro-dissected from Asian patients, *TP53*, *NRAS*, three NF-κB pathway genes (*CYLD*, *TRAF3* and *NFKBIA*), *HLA-A* (the major histocompatibility complex (MHC class I gene), and the transcriptional regulator *MED12L* were all found to be somatically mutated above the baseline level in the pan-Asian population. A higher incidence of gene aberration in NF-κB signaling pathways (~65%) was recently demonstrated in a comprehensive whole-genome sequencing study (Bruce et al., 2021). Ultimately, the aberrant regulation of the NF-κB pathways is significant in NPC tumorigenesis, such as through inducing inflammation, promoting cell survival and proliferation, reprogramming cellular metabolism and activating EBV latent genes including *BARTs*. As recently shown, LMP1 induces SSTR2 via the NF-κB pathway in NPC (Figure 4), which helps to explain the relevance of SSTR2 in exploring diagnosis, immunohistochemistry, imaging methods and treatment options in NPC [5].

## 6. The Role of SSTR2 in Imaging and Targeted Therapeutic Approaches

### 6.1. The ^68^Ga-Dotatate Imaging and SSTR2

Somatostatin analogues such as gallium-68 DOTATATE PET/CT have largely replaced indium-111 pentetreotide SPECT-CT and have demonstrated superiority to SPECT, conventional somatostatin receptor scintigraphy and diagnostic CT in the diagnosis, staging and follow up of NETs [65]. Furthermore, 68Ga-DOTATATE PET/CT detects malignant lesions with greater consistency than indium-111 pentreotide, particularly in the lymph nodes [66]. This superiority is greater still in the case of SSTR2-expressing cells as 68Ga-DOTA-octapeptides were shown to bind SSTR2 100 times more avidly than indium-111 pentetreotide [67]. SSTR2 therefore shows potential as a diagnostic NPC biomarker when coupled with appropriate imaging techniques as demonstrated by a recent clinical trial published in 2021 as part of a larger study by Lechner et al., which found a significant correlation of SSTR2 expression with in vivo 68-DOTA-peptide uptake in 12 NPC patients [5]. This built on research by Khor et al. conducted in 2015 which demonstrated 68Ga-DOTA-peptide PET/CT as an effective biomarker for newly diagnosed undifferentiated NPC [68]. There are additional benefits to 68Ga-DOTATATE PET/CT beyond its superiority for tumor detection. Images are of better quality and patients are exposed to lower radiation dosimetry than 111In-DTPA-octreotide for a whole-body scan and, as shown by Walker et al. in 2013, examination time is shorter by an entire day thanks to its 2-h completion time [69].

### 6.2. Treatment Options

The American Cancer Society details treatment options of NPC by disease stage [70]. The mainstay of treatment for stages 0 and I is radiotherapy to the nasopharynx and prophylactic cervical lymph node radiotherapy. Patients with stage II, III and IVA NPC will receive radiotherapy and chemotherapy, most commonly cisplatin alone or cisplatin with fluorouracil (5-FU). The addition of concomitant chemotherapy to radiotherapy significantly improves survival in patients with locoregionally advanced nasopharyngeal carcinoma. This was confirmed by the first analysis to examine the effect of concomitant chemotherapy with and without adjuvant chemotherapy as distinct groups, a large 2015 study by the Meta-Analysis of Chemotherapy in Nasopharyngeal Carcinoma (MAC-NPC) Collaborative [71]. Crucially, systemic therapies play a key role in the management of NPC due to the large proportion of individuals, who present with distant metastases.

Standard-of-care consists of radiotherapy and chemotherapy, as well as surgery when possible and, most often, in the salvage setting. Late-stage patients are treated with systemic chemotherapy. Radiotherapy is used later on the nasopharynx and cervical lymph nodes if there is a good response to chemotherapy. If the initial chemotherapy appears unsuccessful, a second regimen of different chemotherapeutic agents is used with the potential additions of the monoclonal antibody cetuximab, nimotuzumab and immunotherapy [72,73]. Recurrent and metastatic disease is amenable to systemic platinum-based therapies, which are reserved for these specific conditions [4]. Despite its ability to cause tumor cell death, platinum-based chemotherapy may sensitize these cells to radiation-induced DNA damage and potentiate HMGB1/ATP release [74].

Recently, novel approaches to delivering targeted therapy are being explored. For example, Liu et al. developed a form of targeted NPC therapy through the delivery of chemoradiotherapeutics via folate-functionalised lipid nanoemulsion (LNE) [75]. Soy lecithin nanoemulsion of doxorubicin (Dox) and yittrium-90 (90Y) was prepared using nanoprecipitation via ultrasound homogenisation then folate conjugation. The conjugate FD-Dox + 90Y-LNE, formed by incorporating 90Y into folate decoration over the Dox-LNE surface was shown to induce necrosis and hemorrhage of the cancerous cell line CNE1 and led to reduced tumor volume. The authors concluded in their 2017 trial that FD-Dox + 90Y-LNE may be an efficacious and cost-effective therapy for NPC.

### 6.3. Radionuclide Therapy

Peptide receptor radionuclide therapy (PRRT), a form of targeted radiotherapy, is delivered by somatostatin analogues labelled with β-emitting radionuclides. PET imaging techniques for SS-R-expressing tumors use somatostatin analog peptides such as the DOTA-conjugated peptides were discussed earlier in this review, with roles in the diagnosis, staging and assessment of response to treatment. These peptides, such as octreotate or octreotide, can be chelated with cytotoxic nuclides such as Lutetium-177 or Yttrium-90, or a combination of both, for PRRT. They have proved an effective treatment for inoperable or metastasised NETs, for which few other treatment options exist. Essen et al. found PRRTs to be more effective than chemotherapy in gastroenteropancreatic neuroendocrine tumors (GEPNETs). A 177-Lu-octreotate conferred a survival benefit of several years, and a median response time to treatment of 40 months compared to 30 months with 90Y-DOTATOC [76]. A 2015 review article by van der Zwan et al. noted an average of 15–35% objective response rates of inoperable or metastasised GEPNETs with PRRT. It also purported improvements in anti-tumoral efficacy through delivering combination therapy of PRRT with radiosensitizing chemotherapeutic agents or through combining 90Y and 177Lu [77]. To date, the efficacy of PRRT in the management of NPC has only been described in case reports [78]. Therefore, future studies should be conducted to investigate whether similar outcomes can be achieved in NPC as with other SSTR2-expressing NETs.

### 6.4. Immunotherapy

EBV-associated malignancies, including NPC, have a high level of PD-L1 (PD-1 ligand) expression—therefore, PD-1/PD-L1 immunotherapies are actively being explored and have shown potential efficacy in refractory/metastatic NPC and other malignancies [79]. In NPC, PD-L1 expression is overexpressed in 50–80% of tumors [80]. The EBV oncoprotein LMP-1 and the NF-κB pathway are again both of significance in the PD-1/PD-L1 immune-checkpoint axis, and so this seems a timely moment to remind the reader of the ability of LMP-1 to induce SSTR2 via the NF-κB pathway [81]. The cooperation of LMP-1 and IFN-γ pathways serves to regulate PD-L1 where PD-L1 expression is driven by LMP-1. This upregulation by LMP1 is also associated with the activation of STAT3 and AP-1 in addition to NF-κB signaling, with the overall effect of tumorigenesis through altering critical proteins involved in proliferation, apoptosis and cell invasion [82,83,84].

With the potential efficacy of PRRT discussed above and the prevalence of PD-L1 overexpression in NPC, an additional approach of interest is the combination of PRRT with immunotherapy. This is currently being explored at other sites, including an ongoing phase II trial of pembrolizumab and PRRT in NETs and liver metastases (NCT03457948). While the literature is sparse, given the role of radiation in activating the immune system, it is conceivable that the two modalities may synergize [85]. Indeed, a recent case report of a patient with Merkel Cell Carcinoma previously refractory to PD-L1 immunotherapy demonstrated excellent outcomes with the combination approach [86]. However, this has yet to be explored in NPC.

### 6.5. SSTR2 Targeted Therapies

Over-expression of somatostatin receptors in NETs is well-known and as such the role of synthetic somatostatin analogs such as octreotide and lanreotide in their treatment. Octreotide LAR is FDA-approved for the control of carcinoid symptoms, while lanreotide is approved for its anti-proliferative effect on tumors despite their seemingly interchangeable roles in clinical prescribing [59]. The cytostatic ability of SSTR2 agonism through lanreotide is strongly associated with prolonged progression-free survival and in acromegaly patients, octreotide and lanreotide can achieve rapid and long-term control of hormonal hypersecretion in 50–70% cases [87]. However, its overall anti-proliferative efficacy is limited to tumors with proliferation rates below 10% [88]. Given that SSTR2 over-expression was established in NPC, it may have seemed prudent to consider these agents in its treatment. Lanreotide and octreotide have both been shown to induce upregulation of interleukin signaling and cell senescence pathways [87]. However, they did not demonstrate efficacy in vitro and in vivo, likely due to the fact that proliferation rates of NPC cells are very high [5].

Hence, other targeted therapies, such as antibody-drug conjugates (ADC) and monoclonal antibodies, hold promise as effective treatment options. Recent work has demonstrated the potential safety and efficacy of SSTR2-targeted therapies for the management of NETs [89,90]. PEN-221 is the first pentarin (pen = penetrate, tar = target) cytotoxic conjugate targeting SSTR2 [91]. It consists of the microtubule-targeting agent DM1 linked to the C-terminal side chain of Tyr3–octreotate [89]. Its efficacy was demonstrated in SSTR2-expressing xenograft models of NETs, including small cell lung cancer [92]. As such, PEN-221 is currently being investigated in a phase 1/2a clinical trial for NET patients in the UK and US [93]. Another ADC, targeting SSTR2 and carrying the cytotoxic payload, is monomethyl auristatin E which has demonstrated efficacy in vivo and in vitro [90].

With the observed overexpression of SSTR2 in a large proportion of NPC, SSTR2-targeted therapies may also prove to be effective in the management of this malignancy. Indeed, the safety and efficacy of PEN-221 were recently demonstrated in NPC, with an anti-tumor effect superior to the previously described SS-R agonists in mice [5]. Importantly, the development and implementation of targeted therapies may serve to mitigate treatment-related morbidities associated with standard-of-care. Moreover, with a lack of effective treatment options for metastatic or recurrent disease and the large proportion of individuals, who present with systemic disease, novel treatment approaches are needed. Therefore, it does not seem premature to consider additional studies and protocols utilizing SSTR2-targeted approaches for imaging and therapies, particularly in the recurrent or metastatic setting. The first step would be to evaluate the tumor specimen from the nasopharynx or other regional/distant sites for the expression of SSTR2 on immunohistochemistry. If positive for SSTR2, the patient may be offered 68Ga-DOTA-peptide imaging. High uptake of 68Ga-DOTA-peptide could suggest that the patient may be a candidate for a trial on SSTR2 receptor-targeted radionuclide therapy (Lutetium-177, Yttrium-90) and targeted pharmacological therapy. Future clinical trials investigating the efficacy of this approach are warranted.

### 6.6. Prototype Protocol for a Future Management Option of Treatment-Refractory EBV-Positive NPC

**Figure 5:**
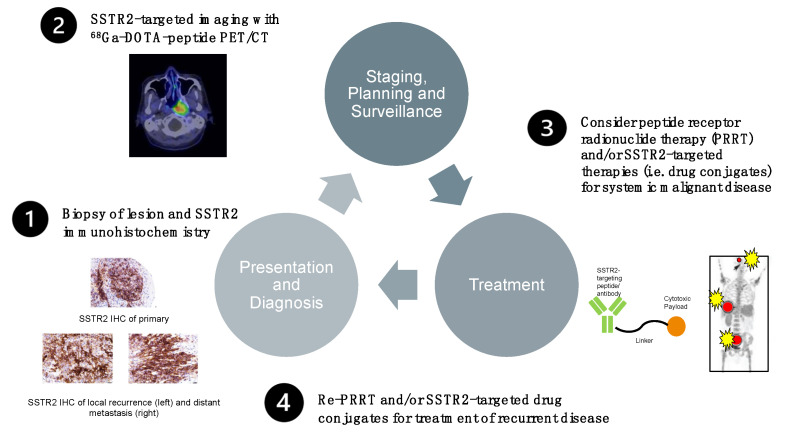
Prototype protocol for future management options for treatment-refractory EBV-positive NPC following initial routine diagnosis of disease. The pathway involves determining the SSTR2 status of a biopsy through immunohistochemistry. Subsequent SSTR2-targeted imaging and therapy may then be offered to eligible patients.

Based on the above, we present a prototype protocol for potential future management options for treatment-refractory EBV-positive NPC following initial routine diagnosis of disease. The pathway involves determining the SSTR2 status of a biopsy through immunohistochemistry. Subsequent SSTR2-targeted imaging and therapy may then be offered to eligible patients. The outline is illustrated in Figure 5.

## 7. Conclusions

SSTR2 is a druggable target in primary, recurrent, and metastatic NPCs and was shown to be overexpressed via activation of the NF-κB pathway through EBV-associated LMP1 or genetic alterations, to which the establishment of latent EBV infection contributes. SSTR2-targeted imaging such as DOTATATE PET/CT may play a future role in the improved diagnosis, staging and surveillance of SSTR2-positive NPCs. Moreover, the prognostic value of SSTR2 has been well documented. Here, we provide a rationale for routine testing of SSTR2 status in NPC patients for prognostic purposes, for exploration of the value of 68Ga-DOTA-peptide PET-CT imaging for NPC diagnosis and surveillance and for offering a theragnostic treatment for advanced and recurrent NPC.

## Data Availability

No new data were created or analyzed in this study. Data sharing is not applicable to this article.

## Supplementary Materials

The following are available online at https://www.mdpi.com/article/10.3390/cancers13194944/s1, Figure S1: NPC Incidence per 100,000 derived from epidemiology data from the World Cancer Research Fund.
