# Supplementary figures and images for: SSTR2 in Nasopharyngeal Carcinoma: Relationship with Latent EBV Infection and Potential as a Therapeutic Target
Source: Cancers (Basel). 2021 Sep 30;13(19):4944. doi: 10.3390/cancers13194944 (PMC8508244; doi:10.3390/cancers13194944)

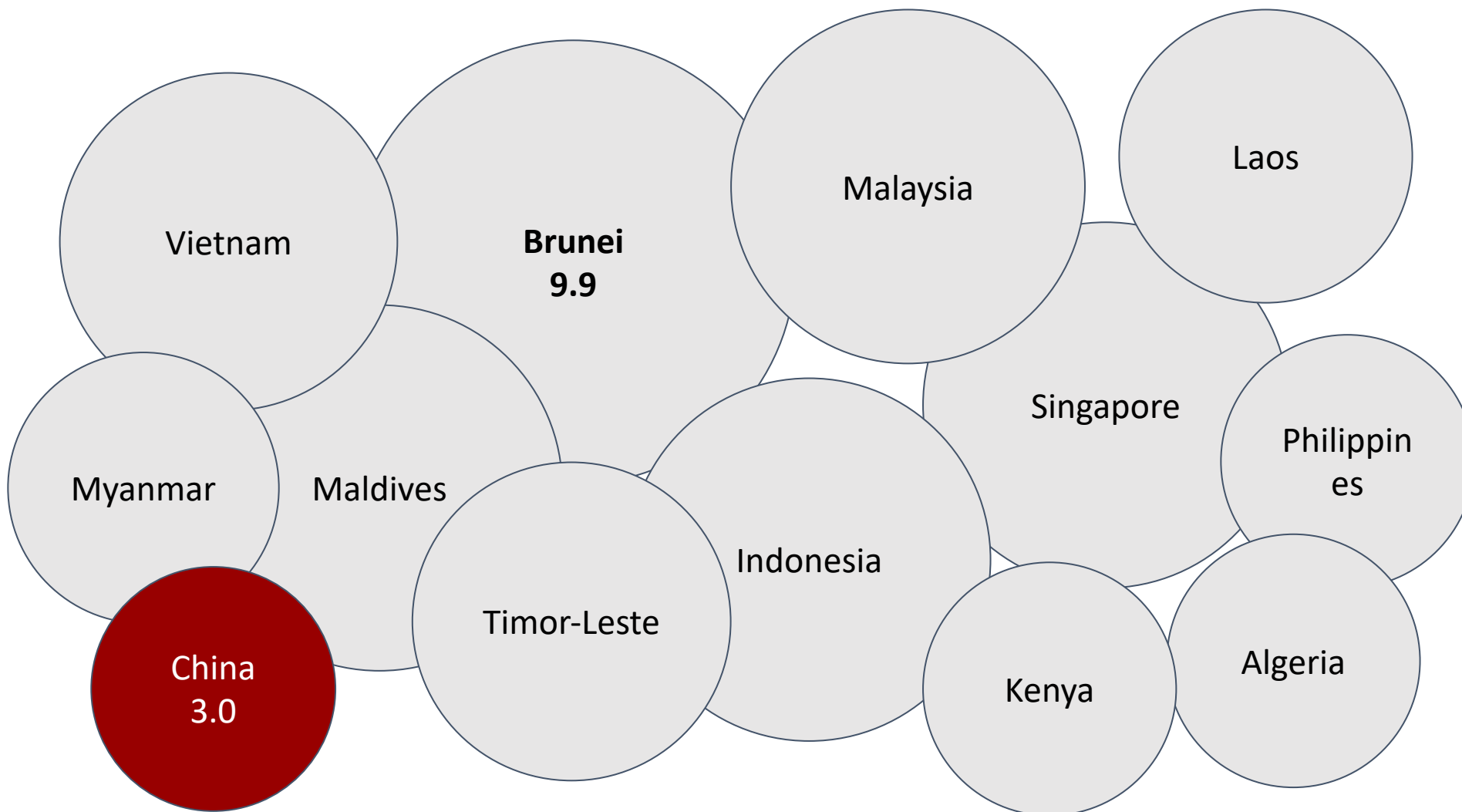

Figure S1: NPC Incidence per 100,000 derived from epidemiology data from the World Cancer Research Fund.

Supplement: Supplementary file 1 [file cancers-13-04944-s001.zip › cancers-1340230-supplementary.pdf]
